# Case Report: Co-occurrence of Duchenne Muscular Dystrophy and Frontometaphyseal Dysplasia 1

**DOI:** 10.3389/fped.2021.628190

**Published:** 2021-02-26

**Authors:** Jaewon Kim, Dong-Woo Lee, Ja-Hyun Jang, Myungshin Kim, Jisook Yim, Dae-Hyun Jang

**Affiliations:** ^1^Department of Rehabilitation Medicine, College of Medicine, Incheon St. Mary's Hospital, The Catholic University of Korea, Seoul, South Korea; ^2^Department of Laboratory Medicine and Genetics, Samsung Medical Center, Sungkyunkwan University School of Medicine, Seoul, South Korea; ^3^Department of Laboratory Medicine, College of Medicine, The Catholic University of Korea, Seoul, South Korea

**Keywords:** Duchenne muscular dystrophy, frontometaphyseal dysplasia 1, X-linked genetic diseases, FLNA gene mutation, genetic disease

## Abstract

Herein, we present a rare case of co-occurring Duchenne muscular dystrophy (DMD) and frontometaphyseal dysplasia 1 (FMD1), two different X-linked diseases, in a 7-year-old boy. He presented with proximal muscle weakness and elevated creatine phosphokinase levels. A multiplex ligation-dependent probe amplification study of *DMD* revealed the *de novo* duplications of exons 2–37, thereby confirming the diagnosis of DMD. Initial evaluation revealed atypical features, such as facial dysmorphism, multiple joint contractures, and severe scoliosis, at an early age. However, these were overlooked and were assumed to be atypical manifestations of DMD. Then, the patient's maternal cousin was diagnosed with FMD1 with pathogenic missense variant in *FLNA* (NM_001110556.2: c.3557C>T/p.Ser1186Leu). A family genetic test revealed that the patient and his mother had the same pathogenic variant in *FLNA*. The patient's atypical manifestations were considered symptoms of FMD1. Therefore, if one disease does not fully explain the patient's clinical features, an expanded genetic study is needed to detect coincidental disease.

## Introduction

Duchenne muscular dystrophy (DMD) is characterized by progressive muscle weakness and atrophy, and it affects ~1 in 3,500−5,000 males (1). DMD is caused by pathogenic variants in *DMD* on chromosome Xp21, and over 2,000 pathogenic variants have been determined. Approximately 55–65% of cases are caused by exon deletions, 30% by single-base variants or small deletions or insertions, and 5–15% by exon duplications (2–5). Individuals with this condition usually experience progressive wasting of the skeletal, respiratory, or cardiac muscles, resulting in death due to cardiac or respiratory compromise (6).

Frontometaphyseal dysplasia 1 (FMD1) is a rare genetic disorder caused by pathogenic variants in *FLNA* on chromosome Xq28 and is characterized by various skeletal development abnormalities. Worldwide, only a few dozen patients have been diagnosed with FMD1 to date. This condition has >10 pathogenic variants, with most being single-base variants (7). Herein, we describe a rare case of co-occurring DMD and FMD1, two different X-linked genetic disorders, coincidently diagnosed in one patient.

## Case Presentation

**Figure 1:**
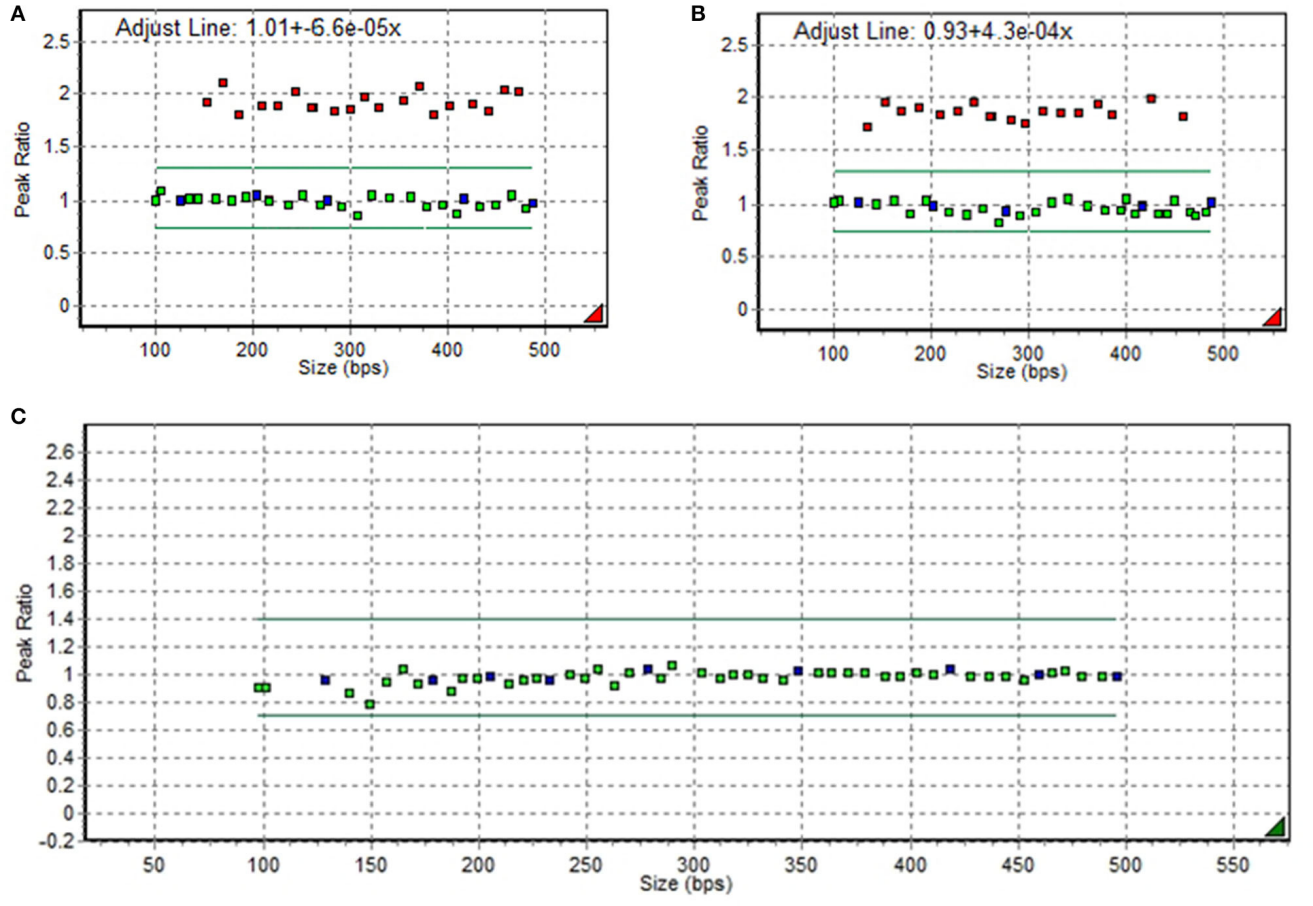
Duplications of exons 2−37 in *DMD* on multiplex ligation-dependent probe amplification electropherogram in the proband **(A,B)** and in his mother **(C)**.

A 7-year-old boy visited the neuromuscular disease clinic of our institution with proximal muscle weakness and multiple contractures of the upper and lower limb joints. There was no significant family history. The patient presented with a waddling gait and Gower's sign. However, he could walk independently and go up the stairs with assistance. The manual muscle test showed grade 3/5 for proximal muscles and 4/5 for distal muscles. Whole-spine radiography revealed scoliosis with a Cobb's angle of 25°. According to laboratory tests, the patient's creatine phosphokinase (CPK) level increased to ~10,000 U/L (reference range, <250 U/L). To check for DMD, a multiplex ligation-dependent probe amplification study of *DMD* was conducted using a kit (MRC Holland, the Netherlands) with two probes (P034-A2 and P035-A1). Results revealed duplications of exons 2–37, which is expected to be out-of-frame (Figures 1A,B). Hence, the proband was diagnosed with DMD. The carrier test result of his mother revealed normal *DMD* without any pathogenic variants (Figure 1C).

Based on physical examination, the patient showed facial dysmorphism with hypodontia, prominent supraorbital ridge, broad and depressed nasal ridge, and micrognathia. Other than muscular dystrophy, various features, such as multiple joint contractures and facial dysmorphism, were atypical manifestations of DMD. The patient received regular steroid and physical therapy. However, multiple joint contractures worsened over time, and 4 years after diagnosis, at 11 years of age, he started using a wheelchair. The patient was subsequently lost to follow-up.

After 3 years, another 13-year-old boy with an appearance similar to that of the current patient visited the neuromuscular disease clinic of our institution due to multiple contractures and facial dysmorphism. Using multigene panel sequencing for skeletal dysplasia, the patient was diagnosed with FMD1 caused by *FLNA* located at chromosome Xq28. The missense *FLNA* variant, which was previously reported as pathogenic variant was identified (NM_001110556.2: c.3557C>T /p.Ser1186Leu) (8, 9). Based on family history, the two children were identified as maternal cousins.

When the DMD patient was 15 years old, we called up the patient and requested him to visit our neuromuscular disease clinic to assess the pathogenic variant of *FLNA*. His joint contractures and scoliosis had worsened (Cobb's angle increased from 25° to 51°), and he was wheelchair-bound due to progressive weakness, joint contracture, and dyspnea. The strength of both shoulder girdles was graded 2/5; upper limbs, 3/5; proximal lower limbs, 2/5; and distal lower limbs, 3/5, and CPK level was 3,156 U/L.

The patient underwent a Sanger sequencing study for *FLNA*. This study revealed that he had a hemizygous missense variant (NM_001110556.2: c.3557C>T), which is the same variant observed in his cousin. Finally, the patient was diagnosed with FMD1 caused by *FLNA* missense variant (c.3557C>T) inherited from the asymptomatic maternal carrier and, coincidently, DMD caused by *de novo DMD* exons 2–37 duplications.

**Figure 2:**
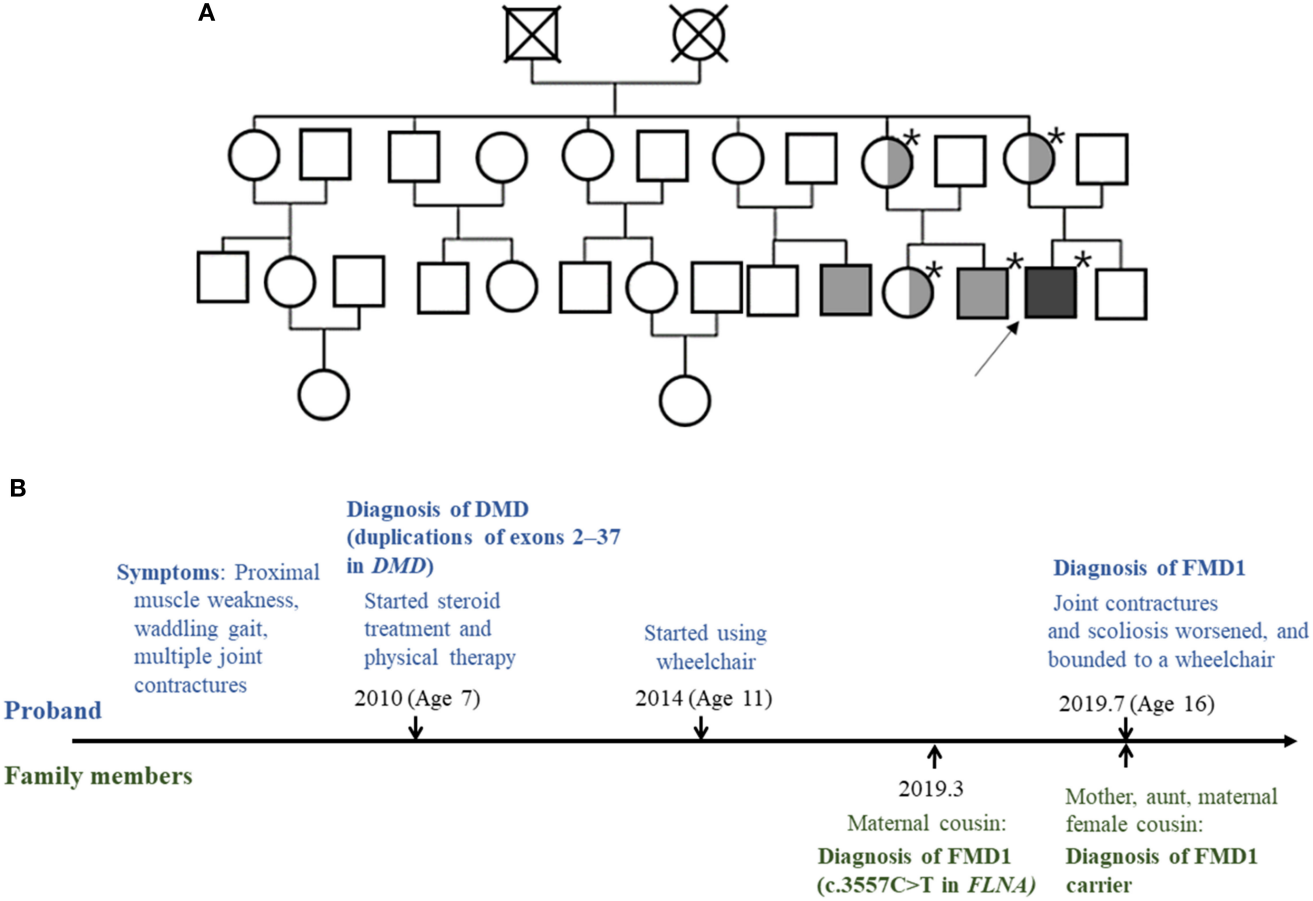
**(A)** Pedigree of the family. The squares and circles represent male and female family members, respectively. The asterisk indicates people tested for the *FLNA* pathogenic variant. Light gray color represents family members with the FMD1 phenotype. Symbols divided into halves indicate heterozygous carriers of FMD1. Dark gray color represents the co-occurrence of FMD1 and DMD. The arrow indicates the proband. **(B)** Timeline of the clinical presentation and molecular diagnosis of the proband and his family members.

**Figure 3:**
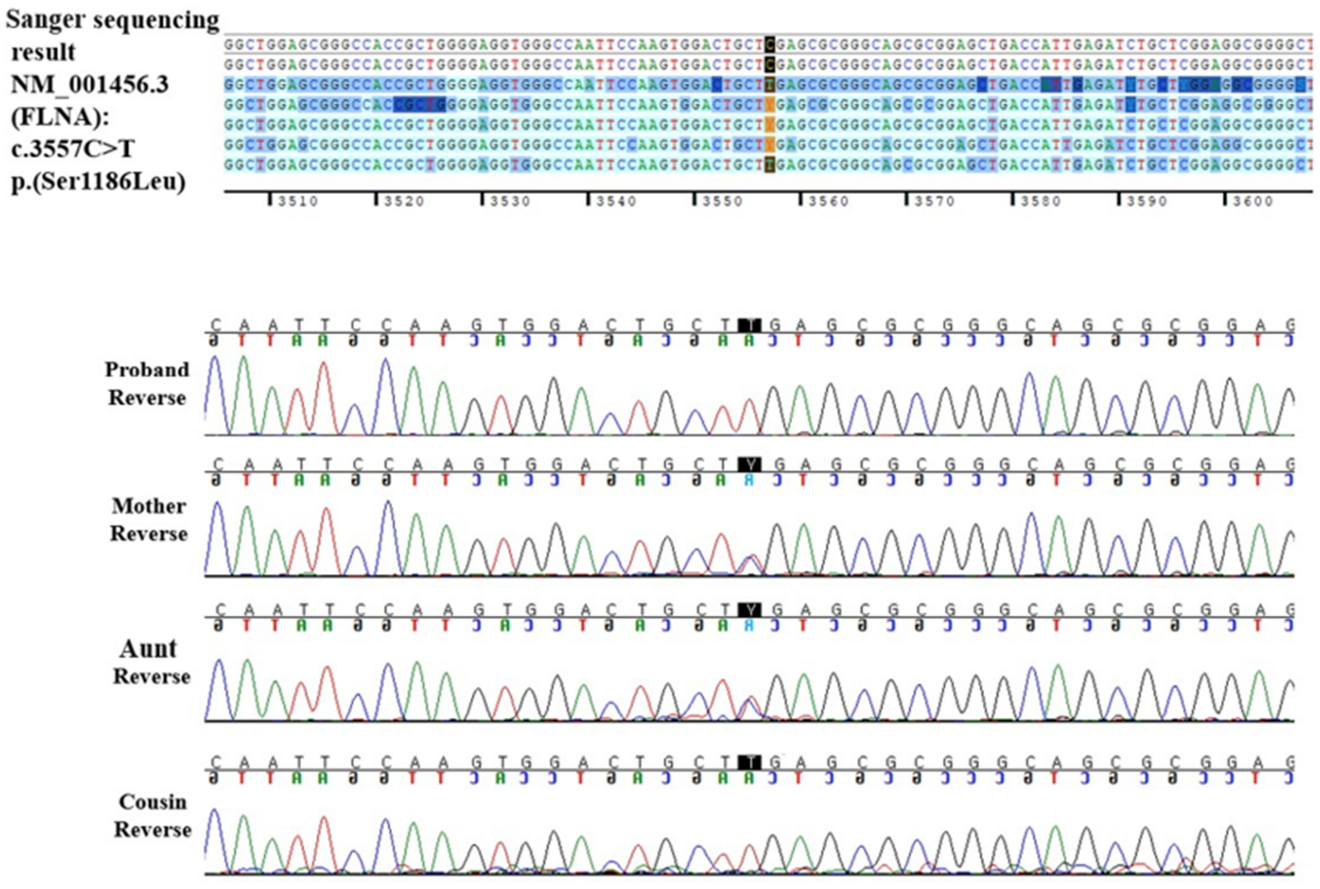
*FLNA* reverse DNA sequencing chromatogram of the proband and his family.

Through family testing, we found that the proband's mother, aunt, and female cousin also had the same pathogenic heterozygous variant of the *FLNA*. None of them showed definite phenotype of FMD1 (Figures 2A,B, 3).

Since the patient's cousin had Chiari I malformation and syringomyelia, brain and whole-spine magnetic resonance imaging was conducted and Chiari I malformation was detected. Additionally, head computed tomography scan revealed craniosynostosis similar to his cousin.

## Discussion

Herein, we report a rare case of co-occurring DMD caused by *DMD* exons 2–37 duplications and FMD1 caused by *FLNA* pathogenic variant (NM_001110556.2: c.3557C>T/p.Ser1186Leu) in a patient. The patient presented with atypical DMD characteristics, such as severe scoliosis, joint contractures, facial dysmorphism, Chiari I malformation, and craniosynostosis. Joint contracture is a common complication of DMD; however, in this case, the patient had developed severe joint contracture at an early age (10).

There have been few cases in which DMD alone induced facial dysmorphism. In a previous case, one patient with DMD presented with facial dysmorphism and craniosynostosis. However, only *DMD* gene-targeted sequencing was conducted, and the presence of other genetic abnormalities was not validated (11). In our case, the presence of multiple contractures and facial dysmorphism, which are not indicative of DMD, was only explained after the diagnosis of FMD1.

This patient presented with two genetic diseases that cause musculoskeletal abnormalities. Hence, whether defective dystrophin and filamin A had interactive or synergic effects was not evaluated in our study but should be considered in the future.

DMD is an X-linked recessive inheritance disorder caused by pathogenic variants in *DMD* that encodes for dystrophin protein, which acts as a connector of the cytoskeleton of muscle fibers and extracellular matrix. These variants help in muscle fiber stabilization during muscle exertion. In the absence of normally functioning dystrophin, progressive muscular damage occurs and regeneration is restricted. Muscle fibers are subsequently transformed into fibrotic or adipose tissues, thus causing pseudohypertrophy (12). In DMD, musculoskeletal complications include progressive muscle degeneration and contracture, resulting in postural compensation or deformities including scoliosis (13). In the later stages, the involvement of cardiac and respiratory muscle becomes apparent. In general, patients require ventilation support during their 20s, and premature death usually occurs during their third or fourth decade of life (14, 15).

FMD1 is a rare genetic disease classified under otopalatodigital syndrome spectrum disorder. It is characterized by skeletal dysplasia, such as multiple joint contractures involving the fingers and/or toes, facial deformity, supraorbital hyperostosis, scoliosis, and sensorineural/conductive hearing loss. The facial features of FMD1 include prominent supraorbital ridge, hypertelorism, and micrognathia (8). In most cases, FMD1 is caused by single-base variants, and all pathogenic variants can maintain the reading frame (9). The *FLNA* comprises 48 exons encoding a 280 kDa filamin A protein. In humans, the filamins are a group of proteins comprising filamins A, B, and C. These proteins are involved in the internal networks of cytoskeleton and filamentous actin rearrangements in the networks in response to mechanical stress. It anchors various transmembrane proteins to the actin cytoskeleton and mediates the signaling processes. Furthermore, filamin A interacts with the glycoprotein-Ibα subunit of the von Willebrand factor receptor, which is associated with cytoskeletal rearrangements, and plays a role in blood vessel and blood clotting (16, 17). Further, filamin C and dystrophin protein were found to have an indirect interaction with each other. However, whether filamin A and dystrophin are correlated or have an interaction with each other is not elucidated (18). Therefore, from a molecular biologic aspect, the presence of these two diseases in one patient is not expected to exert a synergic effect. However, from a clinical aspect, the patient presented with an accelerated deterioration of function, such as gait disturbance, early wheelchair-bound status, severe joint contracture, and scoliosis. If the patient was diagnosed earlier, intensive rehabilitative intervention could have had a positive effect in this case.

The current patient presented with duplications of exons 2–37 in *DMD*, which is an out-of-frame variant, and might have caused DMD (19). In addition, a hemizygous missense variant (NM_001110556.2: c.3557C>T /p.Ser1186Leu) in *FLNA* was previously considered pathogenic, thereby leading to a diagnosis of FMD1.

Until now, there are only a few cases of DMD wherein two or more pathogenic variants occurred simultaneously in the X chromosome. Previous studies have reported Fabry disease (Xq22.1), hemophilia A (Xq28), X-linked myotubular myopathy (Xq28), and oculofaciocardiodental syndrome (Xp11.4) (20–23) (Table 1). *DMD* is the largest human gene known to date, of which one of three pathogenic variants occurs as *de novo*. Hence, in some cases, DMD and other pathogenic variants can coexist in the X chromosome (24).

**Table 1:** Pathogenic variants simultaneously found in the X chromosome in patients with DMD.

**References**	**Gender**	***DMD* variant**	**Combined disease**	**Combined disease gene variant**	**Clinical manifestation**
Takenaka et al. (20)	Male	Exons 46, 47, 50 deletions (inherited)	Fabry disease (inherited)	α-galactosidase A gene c.409delG	Gait disturbance, weakness, CPK↑
Jiang et al. (21)	Female (skewed X-inactivation)	Exons 30–43 deletions (inherited)	X-linked oculo-facio-cardio-dental syndrome (*de novo*)	*BCOR* gene c.1005delC	Muscular hypotonia, weakness, feeding difficulty, CPK↑, congenital anomalies (ASD, cataracts, dental and digital anomalies)
Strmecki et al. (22)	Male	Exons 45–52 deletions (*de novo*)	Hemophilia A (inherited)	Coagulation factor VIII (*F8* gene) Inv22 (distal/type I)	Weakness, CPK↑
Varma et al. (23)	Male	Exons 46–52 deletions (*de novo*)	X-linked myotubular myopathy (inherited)	*MTM1* gene c.1189dupT(p.Tyr397fs)	Weakness, contractures, poor respiratory effort, facial weakness, feeding difficulty
Kim et al. (current case)	Male	Exons 2–37 duplications (*de novo*)	Frontometaphyseal dysplasia 1 (inherited)	*FLNA* gene c.3557C>T(p.Ser1186Leu)	Weakness, gait disturbance, scoliosis, CPK↑, facial dysmorphism, contractures, short stature

*CPK, creatine phosphokinase; ASD, atrial septal defect*.

## Conclusion

Here we report the co-occurrence of DMD and FMD1 in patient, which has not been previously reported to date. Both diseases showed musculoskeletal involvement and X-linked recessive inheritance. DMD occurred *de novo*, and it was incidentally found that FMD1 was a maternal inheritance. In rare cases, two or more genetic disorders can occur concurrently. Therefore, if one disease does not fully explain the patient's clinical features, an expanded genetic study is required to detect coincidental diseases.

## Data Availability Statement

The original contributions presented in the study are included in the article/Supplementary Material, further inquiries can be directed to the corresponding author.

## Ethics Statement

The studies involving human participants were reviewed and approved by Institutional Review Board, Incheon St. Mary's Hospital. Written informed consent to participate in this study was provided by the participants' legal guardian/next of kin.

## Author Contributions

JK: acquisition of data, analysis and interpretation of data, and writing. D-WL: acquisition of data and critical revision of manuscript. J-HJ, MK, and JY: analysis and interpretation of data. D-HJ: study concept and design, acquisition of data, analysis and interpretation of data, study supervision, and critical revision of manuscript for intellectual content. All authors: contributed to the article and approved the submitted version.

## Conflict of Interest

The authors declare that the research was conducted in the absence of any commercial or financial relationships that could be construed as a potential conflict of interest.
