# Supplementary figures and images for: Case Report: Co-occurrence of Duchenne Muscular Dystrophy and Frontometaphyseal Dysplasia 1
Source: Front Pediatr. 2021 Feb 26;9:628190. doi: 10.3389/fped.2021.628190 (PMC7952453; doi:10.3389/fped.2021.628190)

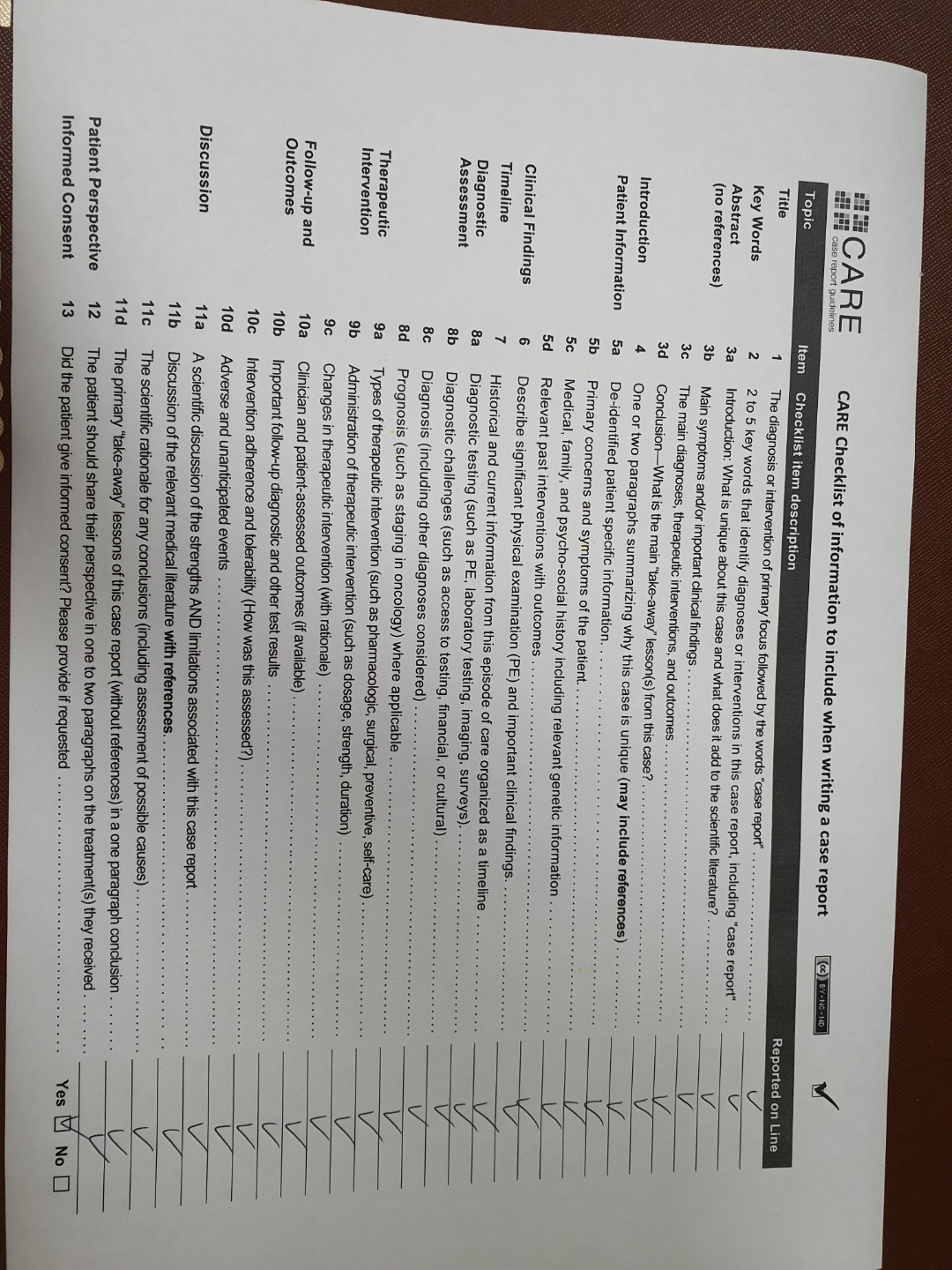

Supplement: Supplementary file 1 [file Image_1.JPEG]
